# Union Medical Society of Northern Indiana

**Published:** 1848

**Authors:** 


### ARTICLE II.

### UNION MEDICAL SOCIETY OF NORTHERN INDIANA.

The following account of the proceedings of the Union Medical Society of Northern Indiana shows by the number of papers read and the spirit of the resolutions passed, that the medical men of that section are taking most efficient measures for mutual improvement and medical reform in that portion of the State.

It is to be hoped that individual members of the profession in the State, as well as other Medical Societies, will promptly lend their aid in procuring the passage of registration laws, in conformity with the recommendations contained in the resolutions. H.

Pursuant to adjournment, the Union Medical Society of Northern Indiana held its fourth Annual Meeting in Goshen on the 2d Tuesday in June, 1848.

Doctors A. C. Jackson and Wm. McBride were, on the recommendation of Censors, admitted as members of this Society.

Doctor Chamberlin read a paper on "the nature and cause of congestion."

Dr. Henkel made a report as Corresponding Secretary and Chairman of the Committee upon quackery.

Dr. E. R. Parks read a paper upon pneumonia.

Dr. John Jackson read a paper on the influence of cold in producing disease, especially in the western country.

Dr. W. J. Matchett read a preamble and set of resolutions on the subject of spurious drugs.

Dr. Latta made a report of the proceedings of the American Medical Association at its last meeting.

On motion of Dr. Henkel, it was

*Resolved,* "That this Society express its satisfaction with the course pursued by Dr. Latta, while representing this body in the American Medical Association; and tender him its thanks for his services, rendered in that capacity."

Drs Chamberlin, Parks, and Jackson were requested to furnish the "North Western Medical and Surgical Journal," with copies of their papers for publication.

Upon motion of Dr. Latta, it was

*Resolved,* That whereas the American Medical Association has recommended the adoption of a law to provide for the registrative of marriages, births, and deaths, and whereas in the opinion of this Society, great political and moral, as well as medical advantages might be derived from such a measure, therefore,

*Resolved,* That the corresponding Secretary be instructed to open a correspondence with the various Medical Societies in this State, soliciting their aid in procuring the passage of proper registration laws.

*Resolved,* That a committee of Five be appointed to have a general charge of the subject.

*Resolved,* That a copy of this preamble and resolutions be forwarded to the Editors of the North Western Medical and Surgical Journal, and that they be respectfully requested to use the influence of their position in bringing the subject before the profession in this State.

Drs. Latta, W. J. Matchett, A. W. Dewy, E. R. Parks, and S. B. Kyler were appointed a committee, under the 3d resolution.

The Secretary presented communications from the New York, Philadelphia, and Baltimore Colleges of Pharmacy:

the proceedings of the State Medical Society of Pennsylvania on the subject of registration laws. Also a letter from Hon. C. W. Cathcart, informing the Society of the passage of a law to prohibit the importation of spurious drugs.

The Society then proceeded to ballot for officers for the ensuing year, which resulted as follows:

For *President*, Dr. E. W. H. Ellis, Goshen, Ind.; *Vice President*, Dr. R. Willard, Oswego, Ind.; *Recording Sec'y*, Dr. M. M. Latta, Goshen, Ind.; *Corresponding Sec'y*, Dr. Paul Henkel, Benton, Ind.; *Treasurer*, Dr. S. B. Kyler, Benton, Ind.; *Censors*, Dr. Chamberlin, Elkhart, Ind.; Dr. J. Jackson, Goshen, Ind.; Dr. Willard, Oswego, Ind.; Dr. Fowler, Bristol, Ind.; Dr. Kyler, Benton, Ind.

Much other business was transacted, which was only interesting to the members of the Society, and therefore would be intruding too much upon your time and space for publication. The Society is in a flourishing condition, and the members are all anxious to see it remain so.

Yours, respectfully, &c.

PAUL HENKEL, *Corres'g Sec'y.*

### A CARD.

Inasmuch as there are many Medical Societies in the State of Indiana with whose officers I am unacquainted, and do not know to whom to address communications in regard to the resolutions passed by the Union Medical Society, at its last meeting, concerning the subject of registration laws, I will deem it a favor if those officers who will see this card would address me on that subject, and others that may be interesting to the Society.

PAUL HENKEL, *Corres'g Sec'y, Benton, Ind.*

